# Augmented Reality Navigation for Extreme Lateral Interbody Fusion with Posterior Instrumentation: Feasibility, Outcomes, and Surgical Technique

**DOI:** 10.3390/bioengineering12111262

**Published:** 2025-11-18

**Authors:** Gabriel Urreola, Matileen G. Cranick, Jose A. Castillo, Hania Shahzad, Allan R. Martin, Kee Kim, Safdar Khan, Richard L. Price

**Affiliations:** Department of Neurosurgery, University of California, Davis, 4610 X Street, Sacramento, CA 95817, USA

**Keywords:** augmented reality, extended reality, spine, extreme lateral interbody fusion, XLIF, prone transpsoas, PTP, lateral lumbar interbody fusion, LLIF, minimally invasive spine surgery

## Abstract

**Background**: Extreme lateral interbody fusion (XLIF) is a minimally invasive spine procedure that traditionally relies on fluoroscopy and neuromonitoring for safe disc space access and instrumentation. Augmented reality (AR) navigation offers real-time anatomical visualization and may reduce fluoroscopy use. This is the first description of applying augmented reality to lateral spine surgery. **Methods**: We conducted a case series of five patients who underwent AR-guided LLIF between May 2024 and July 2025. Surgery was performed in either lateral decubitus or prone transpsoas (PTP) orientation. AR navigation was performed using the Augmedics xvision Spine System, with intraoperative CT–based registration and optical tool tracking. Clinical and operative data, including operative time, estimated blood loss (EBL), length of stay (LOS), radiation exposure, instrumentation accuracy, and postoperative outcomes, were collected and analyzed. **Results**: Five patients (4 female, 1 male; age > 65; BMI range 20.7–37.2) underwent AR-guided XLIF across 8 levels (L2–L5). The mean operative time was 5 h 1 min (range: 2 h 8 min–6 h 45 min), and mean EBL was 94 mL. Mean LOS was 5.85 days (range: 2–10). Mean radiation exposure was 21.73 mGy, significantly lower than published averages for fluoroscopy-guided XLIF (108.6 mGy). At follow-up, all patients reported pain reduction, with 4/5 achieving complete symptom resolution. Instrumentation accuracy was confirmed radiographically in all cases. **Conclusions**: This clinical series demonstrates the first clinical application of AR to lateral lumbar interbody fusion. AR navigation was feasible, safe, and effective, providing accurate disc space access and instrumentation with markedly reduced radiation exposure. These findings support AR as a promising adjunct to improve safety, efficiency, and workflow in lateral spine surgery.

## 1. Introduction

Minimally invasive spine surgery (MISS) techniques have gained traction and evolved as technology has improved. Lateral Lumbar Interbody Fusion (LLIF) is an evolution of MISS done through the lateral of the body to access the disc space to treat pathologies such as degenerative scoliosis, spondylolisthesis, lumbar canal stenosis, and degenerative disc disease [1]. The intra-operative benefits of LLIF include the ability to add in larger footprint cages and avoid intra-abdominal structures compared to when approaching anteriorly [2]. Additionally, LLIF has been shown to reduce hospital stays, lead to faster recovery, and have better pain relief compared to conventional approaches [3,4].

The lateral lumbar interbody fusion (LLIF) is an umbrella term encompassing several techniques, most notably the trans-psoas and pre-psoas (oblique) approaches [5]. The extreme lateral interbody fusion (XLIF) represents a proprietary variation of LLIF that involves traversing the psoas muscle to access the disc space [6,7]. In XLIF, the patient is positioned securely in the lateral decubitus position, and the lumbar spine is accessed through careful dissection through the psoas muscle with the aid of neuromonitoring and tubular retraction systems [6]. Fluoroscopic guidance, typically using both anterior–posterior and lateral views, confirms accurate access, disc preparation, and cage placement. A more recent adaptation, the prone trans-psoas (PTP) approach, also utilizes the trans-psoas corridor but maintains the patient in the prone position [8]. This configuration enables simultaneous posterior instrumentation and has been shown to facilitate greater segmental lordosis correction [9].

One of the challenges with performing an XLIF is avoiding the risk of major nerves of the lumbar plexus, vascular, and peritoneal injury [10]. Traditionally reliant on fluoroscopy and neuromonitoring, this relevant anatomy can be challenging to navigate, especially in severely degenerated or scoliotic patients. Fluoroscopy is utilized to ensure accurate access to the disc space while avoiding critical structures. Neuromonitoring is then employed to minimize the risk of lumbar plexus injury. Advanced imaging and technologies have significantly transformed our ability to visualize anatomy. Augmented Reality (AR) has entered this space as a novel technology by utilizing optical images that are projected into the user’s field of view to create a digital overlay of adjacent anatomy [10]. The most studied use cases are intraoperative pedicle screw placement, but other uses have included spine tumours, rod bending and percutaneous vertebroplasty [11,12,13]. To date, no published reports have described the use of augmented reality (AR) in XLIF procedures. Recently, FDA approval was acquired for AR-based navigation into intervertebral disc spaces, opening the door for novel applications in lateral approaches.

To our knowledge, our study presents the first clinical application of AR navigation in XLIF and PTP procedures. This work introduces a novel intraoperative workflow that integrates AR visualization directly into the surgeon’s field of view, enabling real-time anatomical guidance with minimal dependence on traditional fluoroscopy. The primary purpose of this manuscript is to describe the technical feasibility, workflow integration, and initial clinical outcomes of AR-assisted lateral lumbar interbody fusion. Our early results demonstrate that AR navigation can be safely and effectively implemented during XLIF, offering a promising approach to potentially improve surgical accuracy and reduce radiation exposure to both patients and the operative team.

## 2. Methods

### 2.1. Patient Population

This case series includes clinical data from patients who underwent AR-guided XLIF between May 2024 and July 2025. Five consecutive elective lateral interbody fusion procedures were selected from two attending spine surgeons (RLP and SNK) to undergo AR-enhanced surgery. Patient demographics, including sex, age, BMI, preoperative diagnosis, preoperative pain, and surgical procedure performed, were collected. Clinical and operative variables recorded included operative time, hardware adequacy, evidence of arthrodesis, and radiation exposure. Radiation exposure was quantified as cumulative exposure (mGy), exposure per operated level (mGy), and total fluoroscopy time (minutes:seconds).

### 2.2. Operative Technique

**Figure 1:**
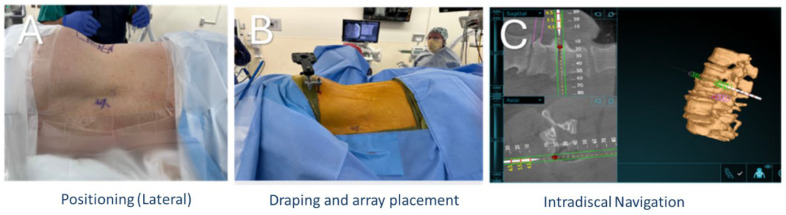
Planning of incision and XLIF trajectory. (**A**) Patient positioned in lateral decubitus with lumbar levels marked. (**B**) Intervertebral disc trajectory mapped, with entry points marked. (**C**) O-arm spin performed to generate a 3D AR image, which was uploaded to the Augmedics headset for anatomical visualization.

**Figure 2:**
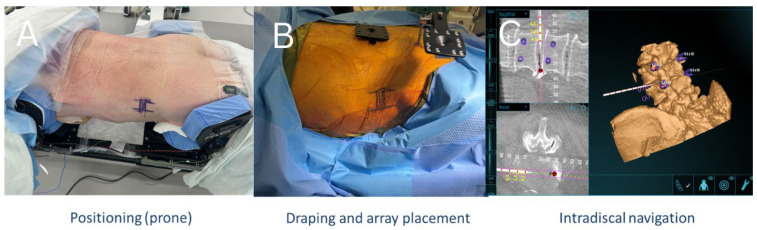
Planning of incision and XLIF trajectory in prone position. (**A**) Patient positioned prone on the operating table with lumbar levels marked. (**B**) Intervertebral disc trajectory mapped, with entry points marked. (**C**) O-arm spin performed to generate a 3D AR image, which was uploaded to the Augmedics headset for anatomical visualization.

A minimally invasive lateral, retroperitoneal, transpsoas approach was performed under general anesthesia with continuous intraoperative neuromonitoring. After induction, patients were positioned either in the lateral decubitus (XLIF) or prone position (PTP) orientation on a ProAxis operating table. Patients were positioned accordingly (Figure 1 and Figure 2).

For each case, and the Augmedics percutaneous reference pin was secured into the iliac wing. The patient marker was affixed to the pin, and the X-link tracking array was placed on the skin (Augmedics, Inc., Chicago, IL, USA). Careful placement of the reference pin and patient are crucial to ensure the ability to perform an intraoperative CT scan. Intraoperative computed tomography (CT) was obtained and registered to the Augmedics augmented reality head-mounted display (AR-HMD). Once calibrated, the AR system provided a heads-up overlay of the patient’s anatomy directly onto the surgical field, enabling real-time visualization of bony anatomy, DICOM-based projections in sagittal and transverse planes, and navigation around bony landmarks and the intervertebral disc space.

**Figure 3:**
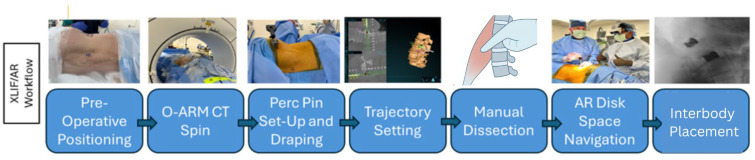
General surgical workflow for AR-guided LLIF. Stepwise depiction of the operative process: patient positioning (lateral or prone), intraoperative O-arm spin with AR registration, placement of the reference pin and tracking array, trajectory planning, manual dissection with navigated probe advancement through the psoas, sequential dilation with triggered EMG, retractor docking, and discectomy with interbody placement under AR guidance.

Using anatomical landmarks, fluoroscopy, and AR navigation, the surgical incision was planned to access the target disc spaces. Subcutaneous tissues were divided, and blunt dissection was carried through the abdominal wall musculature into the retroperitoneal space (Figure 3). Under AR-based navigation, a probe was advanced through the psoas to the disc space (Figure 1C and Figure 2C). Triggered EMG confirmed a safe transpsoas corridor. Once a safe entry zone was established, a tubular retractor was docked orthogonal to the disc space and secured with a shim. AR navigation and fluoroscopy were used to verify positioning at the disc space, and a lateral lumbar interbody fusion was subsequently performed.

A standard discectomy was performed with an operative microscope to aid in direct visualization. Following completion of the discectomy, AR navigation was used to plan interbody placement. Fluoroscopy was applied as needed to confirm accuracy throughout the procedure and during interbody deployment. Hemostasis was obtained and retractors were removed under direct visualization. A stepwise summary of the surgical workflow is provided in Figure 3.

### 2.3. AR Guided Posterior Screw and Rod Placement

Depending on the initial positioning (lateral or prone), the patient was subsequently placed prone for posterior instrumentation. Using AR, the entry point for each pedicle screw was identified, a stab incision was made, and the screw was advanced into the bone under guidance of the 3D overlay displayed in the headset. Percutaneous pedicle screws were placed at the LLIF levels. Fluoroscopy was used to confirm appropriate implant and hardware placement.

### 2.4. Augmented Reality Navigation

The Augmedics xvision Spine System (XVS) was utilized for AR navigation. This platform consists of a head-mounted display (HMD) that projects navigation data directly into the surgeon’s visual field, allowing real-time anatomical visualization without requiring attention to a remote monitor.

Registration was performed using intraoperative CT imaging (O-arm spin). A percutaneous reference pin was inserted into the posterior superior iliac spine (PSIS), to which the patient marker and tracking array were attached, establishing the fixed reference frame. Following calibration, a virtual 3D model of the patient’s anatomy was overlaid onto the surgical field within the HMD. This enabled continuous visualization of bony landmarks, disc spaces, and planned trajectories, with real-time feedback on tool orientation and depth. Instrumentation and interbody work were performed under AR guidance, providing spatial orientation and navigation accuracy with reduced reliance on fluoroscopy.

### 2.5. Surgical Outcome & Statistics

Patients were discharged once deemed clinically stable and were followed postoperatively at 6 weeks and 3 months. Postoperative radiographs were obtained immediately after surgery, as well as at the 6-week and 3-month follow-up visits.

Surgical success was assessed using intraoperative variables, including operative time, estimated blood loss (EBL), intraoperative complications, and length of stay. Additional data collected included the number of levels fused and whether the procedure was performed using a PTP, lateral-to-prone, or all-lateral approach. A few days after surgery, upright radiographs were reviewed to assess the accuracy of interbody and pedicle screw placement.

Clinical outcomes were assessed by comparing postoperative to preoperative pain scores, analgesic use (dose and frequency), and brace utilization. Radiation exposure was measured as cumulative dose, dose per XLIF/PTP level, and total fluoroscopy time (seconds), and compared with 27 conventional XLIF cases performed under fluoroscopic guidance. Radiation data was calculated from intraoperative fluoroscopy records to standardize exposure attributable specifically to navigation. The O-arm spin was excluded from radiation analysis, as it was considered part of routine intraoperative imaging required for AR registration and pedicle screw placement. Descriptive statistics (mean, median, range) were calculated for all variables. Because of the limited sample size, inferential testing was applied only to radiation data, comparing the five AR-navigated cases with 27 conventional XLIF cases using the Mann–Whitney U test. Statistical analyses and figure generation were performed in GraphPad Prism (GraphPad Software Version 10, San Diego, CA, USA), with significance set at *p* < 0.05.

## 3. Results

This patient series includes clinical data from five patients who underwent AR-guided XLIF. Four patients were female, and one was male. All patients were over the age of 65, with BMI values ranging from 20.7 to 37.2. Preoperative diagnoses included adjacent segment disease, degenerative scoliosis, lumbar radiculopathy, spinal canal stenosis, and spondylolisthesis. All patients reported significant preoperative pain that impeded daily living (Figure 1).

**Figure 4:**
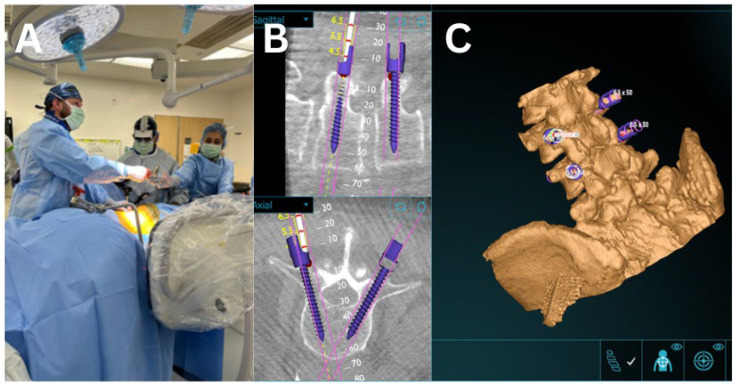
Room setup and AR-assisted workflow. (**A**) Room setup following O-arm CT spin, with two surgeons positioned on the ipsilateral side of the XLIF. The operative surgeon visualizes anatomy on a monitor while the assistant maintains the AR headset for navigation. (**B**) DI-COM view of AR-guided placement of percutaneous pedicle screws. (**C**) Anatomical overlay of vertebral levels with placement of pedicle screws.

**Figure 5:**
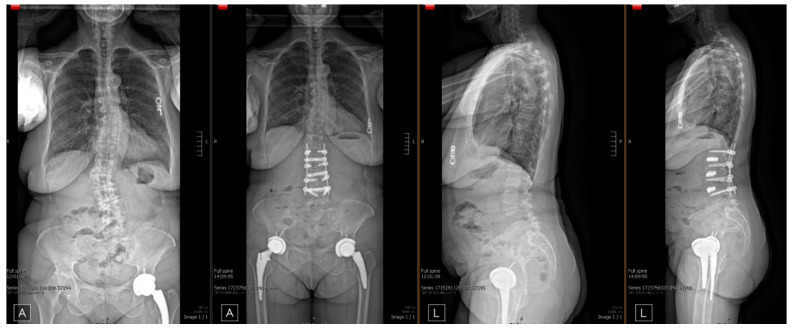
Pre- and postoperative standing radiographs. Example radiographs demonstrating accurate hardware placement and restoration normal spinal alignment following AR-guided L2–5 LLIF with L2–5 PSF.

**Figure 6:**
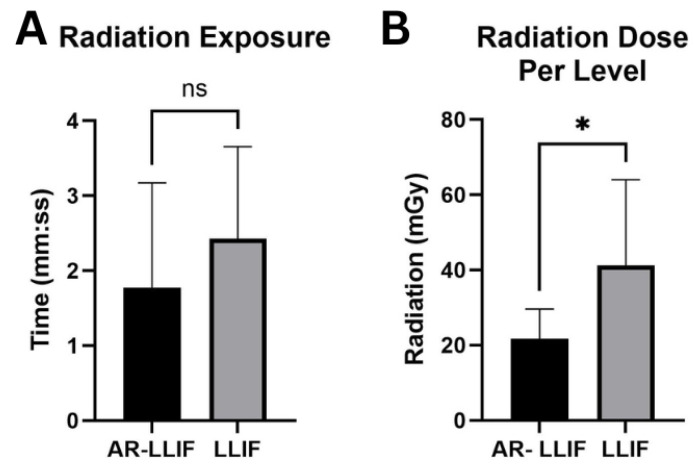
Radiation Exposure: (**A**) Graph demonstrating average fluoroscopy time (s) for AR-guided LLIF compared with traditional LLIF procedures. (**B**) Box plot depicting average radiation exposure (mGy) for AR-guided LLIF, stratified by spinal level, with comparison to traditional LLIF cases. * = Statistically significant difference. ns = Not Significant.

All LLIF procedures were performed between levels L2 and L5 (L2/3: 2, L3/4: 5, L4/5: 1), for a total of 8 levels. Fluoroscopy was utilized to supplement AR navigation to confirm surgical accuracy and proper placement of intrabodies. Most patients subsequently underwent posterior spinal fusion via either a percutaneous technique or a standard midline approach (Table 1) (Figure 4). The average length of stay was 5.85 days (range: 2–10) (Table 2). Average AR-LLIF radiation exposure per level was 21.73 mGy (range: 11.99–33.23), with an average radiation time of 1.77 s (Figure 5) (Supplemental Table S1). By comparison, traditional XLIF cases demonstrated significantly higher average radiation exposure per level (41.5 mGy) (*p* < 0.05) and radiation time (2.18 s) (Figure 6) (Supplemental Table S1). One patient experienced a planned intraoperative complication-a diaphragm injury, that was repaired intraoperatively without further sequelae. Average operating time was 5 h 1 min (range: 2 h 8 min to 6 h 45 min). Estimated blood loss was low across all cases, averaging 94 mL (Table 2).

Follow-up ranged from 1 to 3 months. All patients experienced pain reduction, with four reporting complete resolution of symptoms and three reporting residual but significantly improved pain (Table 2). At the 1-month postoperative visit, hardware adequacy and evidence of successful arthrodesis were confirmed in all cases (Figure 5).

## 4. Discussion

The origins of AR technology for surgical purposes date back to the 1980s, when early attempts were made to superimpose images onto cranial surgical microscopes [14]. Since then, various forms of surgical AR have evolved; however, only in recent years has it been FDA approved and routinely applied in spine surgery [11]. AR was initially validated for its high accuracy and successful implementation in pedicle screw placement. Since then, several platforms including those developed by Augmedics, Novarad, and Medivis have expanded AR applications to a wider range of spinal procedures, including tumour resection, kyphoplasty, and minimally invasive approaches such as transforaminal lumbar interbody fusion (TLIF). Although large-scale clinical validation is still ongoing, early evidence supports several advantages of AR integration, including reduced surgeon attention shifts, hands-free navigation, and decreased radiation exposure. The present study is intended to demonstrate clinical feasibility and provide proof of concept that AR headset–based navigation may improve surgical outcomes in spine procedures while contributing to the growing body of literature. This is the first published study on the application of AR navigation to the LLIF procedure. In line with current trends, our findings support the potential of AR to enhance surgical efficiency and accuracy while reducing radiation exposure [12]. We also believe this work provides a strong foundation for future studies exploring the role of AR in lateral spine surgery approaches.

### 4.1. Radiation Exposure

Using AR, we observed a significant reduction in radiation exposure when performing LLIF compared with both standard XLIF procedures and other interbody fusion techniques. A recent study reported an average radiation dose of 108.6 mGy for XLIF, whereas our average dose per level was significantly lower at 21.73 mGy [15]. In our study, the average radiation dose per level for traditional XLIF procedures under fluoroscopic guidance was 38.47 mGy.

Traditionally, numerous fluoroscopic images are required during instrument placement and disc space localization. With the application of AR, we identified two distinct advantages regarding radiation exposure. First, AR navigation provided rapid and reliable guidance for safe disc space access while simultaneously enabling navigation around critical structures, including the lumbar plexus. Second, accurate percutaneous screw and rod placement was achieved without the need for fluoroscopy at every level. These factors together significantly reduced radiation exposure for both the patient and the surgical team. Additionally, the reduction in fluoroscopy use translated to shorter intraoperative time. Notably, prior studies have also demonstrated that AR-assisted percutaneous screw placement reduces radiation compared to traditional fluoroscopy, consistent with our findings [16].

One limitation of our study is that we did not divide radiation exposure by procedural step (e.g., localization versus pedicle screw placement). Nevertheless, the overall reduction in radiation exposure strongly suggests that AR decreased usage across both components. New registration technologies may further enhance this benefit by enabling AR navigation with intraoperative X-rays instead of CT scans, potentially lowering radiation exposure even further [17]. Future studies with larger sample sizes and multi-institutional designs are warranted to validate our findings and confirm that AR can significantly reduce radiation exposure in spine surgery.

### 4.2. Efficacy, Accuracy and Safety

LLIF efficacy and accuracy are typically assessed using functional, pain, and quality-of-life metrics [2]. Radiological measures such as foraminal height/area, disc height, and evidence of fusion/arthrodesis also serve as important indicators of success [18]. In our series, all patients with documented pain scores demonstrated marked improvements in pain, decreased reliance on pain medications, and the ability to ambulate with minimal to no brace support by one month postoperatively. Clinically, an appropriately placed interbody during LLIF is defined by minimal overhang past each endplate on anteroposterior radiographs, which was achieved in all our cases. Radiographically, our data demonstrated successful fusion/arthrodesis and hardware adequacy in patients who underwent supplementary pedicle screw placement.

Based on accepted definitions of accuracy and efficacy, no mispositioned interbody cages were encountered with AR navigation. Importantly, no patients experienced nerve injury, a complication reported in up to 2.2–19.8% of standard LLIF cases [19]. With respect to pedicle screw placement, no revisions were required for breach or trajectory error.

A key advantage of AR navigation in this approach is the ability to safely access the disc space while traversing critical structures such as the lumbar plexus. The AR head-mounted display enables the surgeon to maintain 3D, direct visualization of the operative field while simultaneously viewing DICOM-based anatomical overlays, allowing hands-free or instrument-guided navigation. This dual-view capability is particularly beneficial in scoliotic or degenerative cases, where disc space access can be challenging; AR overlays enhance accuracy and confidence across multiple levels with variable anatomy. Furthermore, the same system facilitates both disc space access and pedicle screw placement, improving efficiency by integrating these steps into a single navigational platform [20].

Overall, our series demonstrated positive outcomes with no major complications. As AR technology continues to evolve, more advanced anatomical overlays, such as inclusion of the diaphragm, lumbar plexus and adjacent visceral structures, may help further mitigate the risk of intraoperative complications.

## 5. Limitations

A key limitation of our study is the small sample size of 5 patients, which makes interpretation of the impact of AR during LLIF challenging. Despite a limited sample size, the primary purpose of this manuscript is to describe the technical feasibility and workflow of AR in LLIF surgery. While our experience demonstrates the feasibility and potential benefits of AR in XLIF procedures, larger comparative studies are needed to validate these findings. With respect to radiation, our data suggests that AR-guided XLIF may reduce exposure; however, these procedures were often performed in combination with additional interventions, and patient disease severity and risk profiles varied. Future studies should aim to match pathologies and patient characteristics more closely.

Another limitation of our study was the limited assessment of radiographic parameters such as foraminal height, disc height, and evidence of fusion over a longer follow-up period. Our manuscript primarily focused on surgical technique and radiation metrics, but we recognize the importance of incorporating these additional radiographic and clinical measures in future analyses. Fusion status was evaluated up to three months postoperatively; however, we acknowledge that a six-month or longer follow-up period is often a more reliable indicator of fusion. We consider this a meaningful limitation and plan to address it in future studies. This represents the next phase of our ongoing work and will allow for a more comprehensive comparison between AR-guided and conventional XLIF cases.

## 6. Conclusions

The integration of AR technology into spine surgery represents a significant advancement in minimally invasive techniques [14]. Our initial experience with AR-guided LLIF highlights several key advantages over traditional approaches. First, AR navigation reduced reliance on fluoroscopy during lateral access, addressing a longstanding concern in minimally invasive spine surgery [16]. By enabling precise visualization of critical anatomical structures and accurate disc space targeting without repeated imaging, AR has the potential to decrease radiation exposure for both the surgical team and the patient. Second, our series demonstrated successful outcomes across multiple spinal levels (L2–L5), including both primary and revision cases. Third, we observed safety across a wide spectrum of indications—from large deformity cases to revisions and canal stenosis—with no major complications.

While our experience supports the feasibility and potential benefits of AR in LLIF procedures, larger comparative studies are needed to validate these findings. This work introduces a novel AR workflow integrated into the LLIF technique and establishes a foundation for future investigations combining augmented reality with other minimally invasive spine approaches. To our knowledge, this represents the first clinical application of AR navigation to the LLIF procedure and adds to the growing body of literature by demonstrating its feasibility, safety, and potential to meaningfully reduce radiation exposure. In summary, this clinical case series contributes new evidence supporting AR as a promising adjunct in spine surgery, with the potential to enhance surgical accuracy, efficiency, and overall operative safety.

## Figures and Tables

**Table 1:** Clinical features and demographics of 7 patients who underwent AR guided XLIF procedure.

Patient No.	Age	Sex	BMI	Pre-Operative Diagnosis	Procedure(s)
1	75	M	21.9	- Adjacent segment disease L2–L4 - Lumbar Radiculopathy	(1). Hardware Removal (2). L2-5 Revision MIS PSF (3). L2-L4 PTP XLIF
2	66	F	27.4	- Degenerative Scoliosis - L2–L5 Lumbar Stenosis	(1). L2-5 XLIF
3	69	F	20.7	- L3–L4 spondylolisthesis	(1). L3-4 XLIF (2). L2-L5 MIS PSF
4	65	F	27	- L3–L4 spondylolisthesis	(1). L3-4 PTP XLIF (2). L3-4 MIS PSF
5	71	F	34.7	- Spinal Stenosis w. neurogenic claudication	(1). L3-4 XLIF (2). L3-4 PSF
6	64	M	37.2	- Scoliosis of lumbar spine	(1). L2-5 XLIF (2). L2-5 PSF
7	78	M	34.1	- Spinal stenosis without neurogenic claudication	(1). L2-5 PSF (2). L2-5 XLIF

Minimally invasive surgery (MIS); Posterior spinal fusion (PSF).

**Table 2:** Intra-operative and post-operative outcomes.

Patient No.	Surgery Time	EBL	LOS (Days)	Operative Complications	Hardware Adequacy/ Arthrodesis	Pain Score Pre-Op/Post-Op	Current Pain Meds
1	6 h 45 min	200 mL	5	None	Yes/Yes	8/10/0/10	Tylenol prn
2	5 h 38 min	150 mL	10	Planned diaphragm injury	Yes/Yes	-	None
3	5 h 20 min	20 mL	5	None	Yes/Yes	-	Methadone 10 mg BID
4	2 h 8 min	50 mL	6	None	Yes/Yes	6/10/0/10	None
5	5 h 16 min	50 mL	2	None	Yes/Yes	8/10/2/10	Gabapentin 600 mg

## Data Availability

The original contributions presented in this study are included in the article/Supplementary Material. Further inquiries can be directed to the corresponding author.

## Supplementary Materials

The following supporting information can be downloaded at: https://www.mdpi.com/article/10.3390/bioengineering12111262/s1, Supplementary Table S1: Clinical data related to radiation exposure.
