# Supplementary material for: Augmented Reality Navigation for Extreme Lateral Interbody Fusion with Posterior Instrumentation: Feasibility, Outcomes, and Surgical Technique
Source: Bioengineering (Basel). 2025 Nov 18;12(11):1262. doi: 10.3390/bioengineering12111262 (PMC12650056; doi:10.3390/bioengineering12111262)
Supplement: Supplementary file 1 [file bioengineering-12-01262-s001.zip › bioengineering-3941766-supplementary.pdf]

Supplementary Tables

Supplementary Table S1: Clinical data related to radiation exposure

**Patient No.**	**Levels Performed**	**Dose Area Product (Gycm2)**	**Cumulative Dose (mGy)**	**Dose Per Level**	**Radiation Exposure Time (m.ss)**
			AR Guided XLIF Cases [1-5]		
1	2	1351.17	54.94	24.528	3.27
2	3	2212.83	71.6	20.657	3.19
3	1	NA	NA	11.993	1.14
4	1	1054.93	22.94	18.282	1.16
5	1	11216.15	83.05	33.233	0.1
			Fluoroscopy Guided XLIF Cases [8-36]		
6	3	27.00	75.98	25.33	3.9
7	3	37473.40	108.32	36.11	3.7
8	2	57.98	199.88	99.94	3.3
9	1	27.50	62.56	62.56	1.50
10	3	70981.30	163.94	54.65	3.50
11	4	56134.70	168.48	42.12	4.58
12	3	18.91	65.26	21.75	3.3
13	1	12299.80	28.98	28.98	1.6
14	1	12.07	36.47	36.47	1.3
15	3	18199.40	45.96	15.32	3.7
16	3	24.91	56.68	18.89	3.52
17	1	14.15	44.34	44.34	1.20
18	1	5616.90	12.79	12.79	1.34
19	3	64.56	183.24	61.08	3.58

20	1	38.59	94.19	94.19	1.10
21	2	39.27	89.50	44.75	2.39
22	3	30.91	71.67	23.89	3.60
23	3	34.68	90.34	30.11	3.2
24	1	7870.00	17.95	17.95	0.56
25	1	13.82	31.45	31.45	1.50
26	1	12.68	28.60	28.60	1.30
27	1	13.82	31.45	31.45	1.50
28	1	13.74	35.19	35.19	1.10
29	2	37.53	85.38	42.69	4.49
29	1	35.06	86.63	86.63	1.40
30	1	15.38	34.98	34.98	1.80
31	1	19.05	48.72	48.72	1.48
